# Analysis of protein kinase C (*HcPKC*) gene expression and single-nucleotide polymorphisms related to inner shell color traits in *Hyriopsis cumingii*

**DOI:** 10.1186/s12863-022-01085-3

**Published:** 2022-09-09

**Authors:** Mengying Zhang, Xiajun Chen, Jinpan Zhang, Baiying Guo, Jiale Li, Zhiyi Bai

**Affiliations:** 1 Key Laboratory of Freshwater Aquatic Genetic Resources, Ministry of Agriculture and Rural Affairs, Shanghai Ocean University, Shanghai 201306, China; 2 Shanghai Collaborative Innovation Center of Aquatic Animal Breeding and Green Aquaculture, Shanghai Ocean University, Shanghai 201306, China; 3 Fisher Institute of Anhui Academy of Agricultural Sciences, Hefei, China; 4 Shanghai Engineering Research Center of Aquaculture, Shanghai Ocean University, Shanghai 201306, China

**Keywords:** Hyriopsis cumingii, Nacre color, HcPKC, SNP

## Abstract

**Background:**

Protein kinase C (PKC) is a multifunctional serine and PKC can phosphorylate serine residues in the cytoplasmic domain of tyrosinase, thereby regulating the activity of tyrosinase. Activated PKC is bound to the melanosome membrane, and unactivated PKC is free in the cytoplasm of melanocytes. In this study, we study the role of PKC gene in the melanin synthesis pathway and its effect on the color of the nacre of *H. cumingii*.

**Results:**

In this study, a *HcPKC* gene in *H. cumingii* was cloned and its effects on melanin synthesis and nacre color were studied. *HcPKC* was expressed in both purple and white mussels, and the level of mRNA expression was higher in the purple mussels than in white mussels. Strong and specific mRNA signals were detected in the dorsal epithelial cells of the mantle pallial layer, indicating that *HcPKC* may be involved in nacre formation. After SNP association with inner shell color related traits, according to the principle that 0.25 < PIC < 0.5 is medium polymorphism and PIC < 0.25 is low polymorphism, the A + 332G site on the *HcPKC* gene was a site of moderate polymorphism, and the other four sites were low polymorphism sex sites. There was strong linkage disequilibrium among the five loci. A haplotype was constructed and it was found that the frequency of T1 (AGGAA)in the white population was significantly higher than that in the purple population (*P* < 0.05).

**Conclusion:**

The study found that *HcPKC* of *H. cumingii* can be used as a candidate gene related to inner shell color, and some of the SNP sites can be used for molecular-assisted breeding in the spinnaker mussel, providing a reference for cultivating high-quality freshwater pearls.

## Background

*Hyriopsis cumingii* is a unique freshwater mussel in China, which can cultivate high-quality pearls [1]. Mussels of this species produce pearls of high quality in terms of color, luster, and shape [2]. The freshwater pearls produced by purple *H. cumingii* have a very high economic value [3]. Recently, due to overexploitation, habitat loss, and environmental pollution, wild populations have declined significantly and are facing local extinction [2]. Therefore, the need to harvest high-quality freshwater pearls artificially is imminent. However, there are few relevant studies on the mechanisms involved in the formation of shell nacre color, but it is still in its infancy. Studies have shown that in addition to environmental factors, the color of pearls is also affected by the donor and recipient mussels [4]. Some studies suggest that some metal ions can also affect the formation of pearl color [5, 6], and other studies show that pearl color is related to organic pigments, such as porphyrin [7], carotenoids [8], and melanin [9–11], which produce pearls of different colors.

Protein kinase C (PKC) is a lipid- and Ca^2+^-dependent serine/threonine kinase consisting of a single polypeptide chain [12]. Because the structure of each subtype has a certain conservation and specificity, the functions of the subtypes are also diverse [13]. PKC widely exists in animal tissues and cells, is the main mediator of signal transduction pathway, and also plays a role that cannot be underestimated in physiological processes such as cell proliferation and differentiation [14]. In the melanin metabolism pathway, PKC activates the tyrosinase by phosphorylation of its two serine residues [15]. Activated PKC is bound to the melanosome membrane, and unactivated PKC is free in the cytoplasm of melanocytes [16]. The physiological activation of PKC reportedly stimulates melanin production [17], whereas the inhibition of PKC activity or depletion of cellular PKC has been shown to inhibit melanin synthesis [18]. Park et al. [19] paired cultures of primary human melanocytes treated with PKC inhibitors, found that the PKC inhibitor bisindolylmaleimide can reduce skin pigmentation, and demonstrated that the inhibition of PKC-β activity can reduce pigmentation. Jung et al. [20] found that syndecan- 2 overexpression increased the membrane localization of PKCbΙΙ, and that activated PKCbΙΙ associates with the melanosome through RACK1 to regulate melanogenesis.

In this study, a PKC gene (*HcPKC*) was identified in *H. cumingii*, and its full length was cloned. The expression level of the *HcPKC* gene was detected in different tissues. In situ hybridization was used to detect the distribution of mRNA expression in the mantle. Single-nucleotide polymorphism (SNP) mutation sites were detected in *H. cumingii* using *HcPKC* as a candidate gene and correlation analysis was performed with color traits. The molecular markers related to the color traits of the shell nacre were screened and then *H. cumingii* were selected. This selection and breeding process provides basic data for further research.

## Results

### Full-length and sequence analysis of *HcPKC* gene

**Fig. 1:**
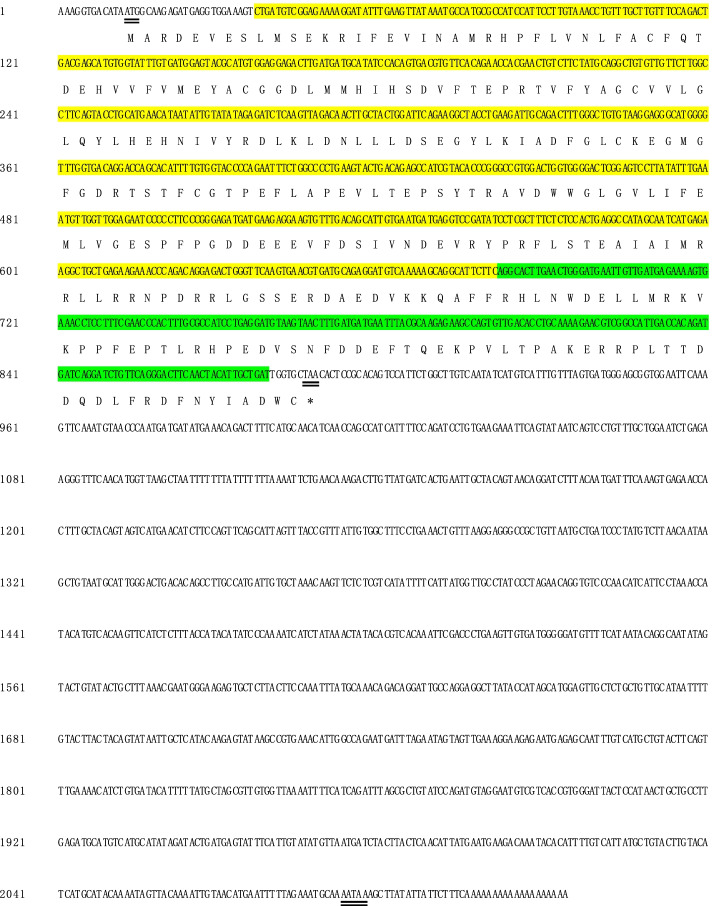
cDNA sequence analysis of the HcPKC gene in H. cumingii. The shaded part is the domain. The start codon, stop codon, and the poly-A tail are underlined. The shaded part of yellow represents S_TKc domains typical of serine- and threonine-specific kinase families. The green shaded part represents S_TK_X domains typical of serine- and threonine-specific kinase families

The full length of the *HcPKC* (GenBank accession MW241548) gene was obtained by 3′ and 5′ RACE cloning. The *HcPKC* gene sequence is 2134 bp in total, of which the 5′-UTR was 12 bp, the 3′-UTR was 1246 bp, and the ORF was 876 bp long, encoding a total of 291 amino acidsThe molecular weight of the mature protein corresponding to the amino acid sequence was 117.04 kDa, and the isoelectric point was calculated as 4.73. S_TKc and S_TK_X domains typical of serine- and threonine-specific kinase families were found. No signal peptide was found (Fig. 1).

### Quantitative gene expression analysis

**Fig. 2:**
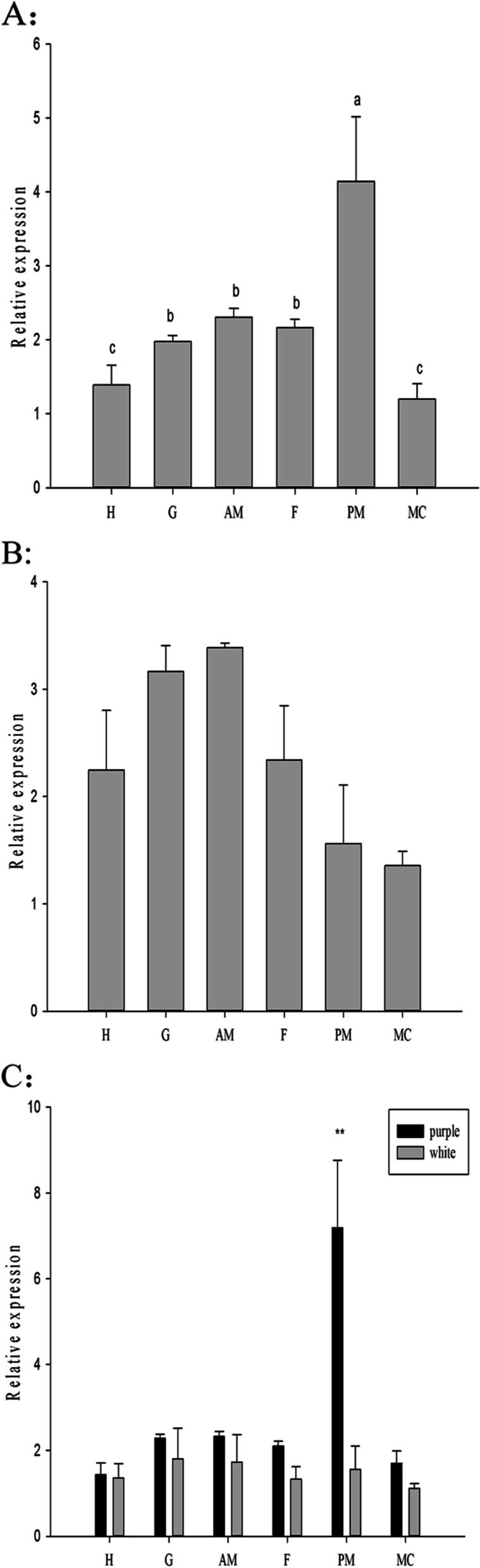
Relative expression level of HcPKC. The relative expression level of PKC in various tissues of purple (**A**) and white (**B**) mussels. Comparison of PKC expression in white and purple mussels (**C**). H: hepatopancreas, G: gill, AM: adductor muscle, F: foot. PM: marginal membrane, MC: central membrane. Data from the qPCR experiments are expressed as the means ± SD (*n* = 6). Bars with different letters indicate significant differences (*p* < 0.05). ** represents a highly significant difference at *P* < 0.01

The relative expression of the *HcPKC* gene in purple and white mussels was detected by qPCR. As shown in Fig. 2, the expression of *HcPKC* in purple mussels was higher than that in white mussels, with an extremely significant difference in the marginal membrane (*P* < 0.01), and no significant difference in other tissues. In purple mussels, the highest expression was in the marginal membrane, and it was significantly different from other tissues (*P* < 0.05). In the white mussel, the highest expression was in the adductor muscle, but there was no significant difference between the tissues.

### In situ hybridization results

**Fig. 3:**
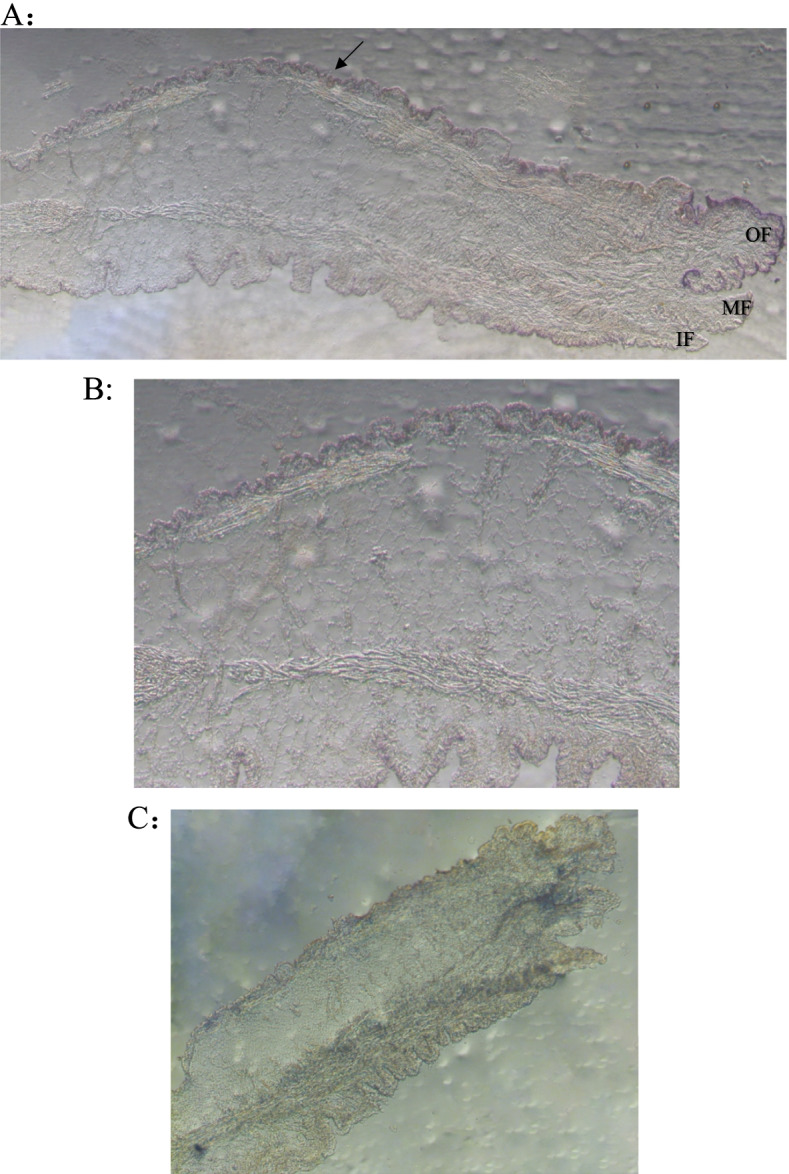
In situ hybridization analysis of HcPKC in the mantle (**A**), the arrow indicates the position of the hybridization signal. **B** is a higher magnification of **A**, **C** is background. IF, inner fold; MF, middle fold; OF, outer fold

The location of the specific expression of the *HcPKC* gene in the mantle tissue was determined by in situ hybridization. The results are shown in Fig. 3, The positive hybridization signal mainly appeared in the dorsal membrane epithelial cells of the outer fold of the mantle (arrow in Fig. 3 A), and no obvious signal was seen in other parts. No positive signal was detected in the negative control group.

### SNP site screening

The samples were amplified with the designed primers, and a total of five SNP sites were found in the amplified fragments. Starting from the ATG start codon, each SNP site is named by the number of bases from the mutation site to the start codon.

### Polymorphism analysis

The *HcPKC* gene was amplified and sequenced from 70 purple mussels and 70 white mussels to screen for the SNP loci. The polymorphic genetic parameters of the five SNP loci of the *HcPKC* gene obtained after the sequencing results were analyzed by software (Table 1). Their observed heterozygosity was in the range of 0.0143–0.0929, the expected heterozygosity was in the range of 0.0624–0.3254, the polymorphic information content(PIC) was in the range of 0.060–0.272, and the effective number of alleles was in the range of 1.0663–1.4799. A 0.25 < PIC < 0.5 was considered moderate polymorphism and PIC < 0.25 was considered low polymorphism, the A + 332G site on the *HcPKC* gene was a site of moderate polymorphism, and the other four sites were low polymorphism locus.

**Table 1:** The polymorphic parameters of five SNP sites in the *HcPKC* gene

	Observed heterozygosity	Expected heterozygosity	Polymorphic information content	Effective number of alleles
Suit	H_0_	H_e_	PIC	N_e_
A + 87 T	0.0214	0.0624	0.060	1.0663
G + 145 T	0.0500	0.1513	0.139	1.1776
G + 217 T	0.0143	0.0953	0.090	1.1050
A + 328G	0.0786	0.1432	0.149	1.1942
A + 332G	0.0929	0.3254	0.272	1.4799

### Association analysis between the SNP loci of the *HcPKC* gene and inner shell color traits

The genotypes of the SNPs found on the *HcPKC* gene were correlated with the inner shell color traits (*L*, *a*, *b*, and *dE*) of 140 mussels (Table 2). The results showed that among the five SNPs in the *HcPKC* gene, there was no significant difference between the genotypes of the three loci A + 87 T, G + 145 T, and A + 328G, and the parameters of the four inner shell color traits. The genotypes of the G + 217 T locus had significant differences in *b* and *dE* parameters (*P* < 0.05) and the genotypes of the A + 332G loci had significant differences in *L*, *b* and *a*, *dE* parameters (*P* < 0.05).

**Table 2:** Association of the five SNP sites of *HcPKC* polymorphisms with nacre color

Locus	Genotype	No	M*L*	M*a*	M*b*	M*dE*
A + 87 T	AA	134	54.17 ± 0.70^A^	3.38 ± 0.22 ^A^	0.36 ± 0.56 ^A^	47.51 ± 0.72 ^A^
	AT	3	54.92 ± 5.70 ^A^	3.81 ± 1.64 ^A^	-1.73 ± 3.62 ^A^	46.16 ± 6.19 ^A^
	TT	3	59.14 ± 4.36 ^A^	2.62 ± 1.49 ^A^	-4.96 ± 2.19 ^A^	41.73 ± 4.42 ^A^
G + 145 T	GG	125	54.44 ± 0.74 ^A^	3.36 ± 0.23 ^A^	0.39 ± 0.58 ^A^	47.20 ± 0.76 ^A^
	GT	7	50.92 ± 2.71 ^A^	4.52 ± 0.85 ^A^	-0.25 ± 2.95 ^A^	50.97 ± 2.90 ^A^
	TT	8	54.98 ± 2.44 ^A^	2.73 ± 0.89 ^A^	-2.24 ± 2.16 ^A^	46.69 ± 2.47 ^A^
G + 217 T	GG	132	54.21 ± 0.70 ^A^	3.42 ± 0.23 ^A^	0.39 ± 0.57^AB^	47.46 ± 0.73 ^A^
	GT	2	41.21 ± 6.74 ^A^	3.13 ± 2.35 ^A^	6.90 ± 3.42^B^	60.89 ± 6.28 ^B^
	TT	6	60.52 ± 3.03 ^A^	2.49 ± 0.90 ^A^	-6.20 ± 2.22 ^A^	40.60 ± 3.25 ^A^
A + 328G	AA	122	54.44 ± 0.74 ^A^	3.31 ± 0.23 ^A^	0.30 ± 0.58 ^A^	47.19 ± 0.76 ^A^
	AG	11	52.57 ± 2.57 ^A^	4.55 ± 0.76 ^A^	0.72 ± 2.07 ^A^	49.30 ± 2.64 ^A^
	GG	7	54.44 ± 2.70 ^A^	2.61 ± 1.03 ^A^	-2.71 ± 2.38 ^A^	47.17 ± 2.73 ^A^
A + 332G	AA	105	55.14 ± 0.78 ^B^	3.07 ± 0.24 ^B^	0.10 ± 0.63 ^AB^	46.44 ± 0.81 ^A^
	AG	13	56.27 ± 2.34 ^B^	3.57 ± 0.71 ^AB^	-2.79 ± 1.68 ^A^	45.19 ± 2.42 ^A^
	GG	22	49.07 ± 1.71 ^A^	4.72 ± 0.65 ^A^	2.47 ± 1.30 ^B^	53.04 ± 1.77 ^B^

*Notes*: Different superscript letters in a column of the same two loci indicate significant difference at *P* < 0.05

### Linkage disequilibrium and haplotype analysis of the SNP loci in the *HcPKC* gene

Linkage disequilibrium analysis was performed on the five SNP loci (Table 3), and it was found that there was a strong linkage disequilibrium between all the loci (D' > 0.75, r^2^ > 0.33). After haplotype construction (Table 4), it was found that T1 appeared more frequently in the white population than in the purple cultivar.

**Table 3:** Linkage disequilibrium analysis of the five SNP sites of the *HcPKC* gene

	A + 87 T	G + 145 T	G + 217 T	A + 328G	A + 332G
A + 87 T	-	1.000	1.000	1.000	1.000
G + 145 T	0.371	-	1.000	0.952	1.000
G + 217 T	0.631	0.588	-	1.000	1.000
A + 328G	0.339	0.827	0.537	-	1.000
A + 332G	0.130	0.350	0.206	0.384	-

*Notes*: The figure above the diagonal represents D', the figure below the diagonal represent r ^2^

**Table 4:** Haplotype analysis of the five SNP sites of the *HcPKC* gene

Haplotype	Sequence	Purple strain(frequency)	White strain(frequency)	χ2(*P* value)
T1	A G G A A	99.00(0.707)	113.00(0.807)	5.379(0.020)
T2	A G G A G	22.00(0.157)	13.00(0.093)	2.681(0.101)
T3	A T G G G	7.00(0.050)	2.00(0.014)	2.879(0.089)

## Discussion

In this study, a *HcPKC* gene was fully cloned in *H. cumingii* and investigated for the first time. The tissue quantification results showed that the expression level of *HcPKC* in the marginal membrane of purple mussels was significantly higher than that of other tissues (p < 0.05). Relevant studies have shown that the outer fold of the mantle is directly involved in the formation of shell nacre [21, 22]. Protein kinase C not only plays a role in the process of melanin synthesis, but also plays a role in other physiological activities, such as nerve and immunity [23], the specific location of *HcPKC* expression in the mantle tissue was determined by in situ hybridization, and a positive hybridization signal mainly appeared in the mantle. The results from the dorsal membrane epithelial cells at the outer fold suggest that *HcPKC* may be involved in the formation of the nacre in *H. cumingii* [24]. Further comparative analysis found a higher expression of *HcPKC* in the tissues of purple mussels than in those of white mussels, and there was a very significant difference in the marginal membrane (*p* < 0.01). Luo et al. [25] found similar results in the phenotypic difference of the *HcCUBDC* gene in white and purple *H. cumingii*, this indicates that the *HcPKC* gene may have a positive effect on the formation of purple nacre.

Studies have shown that the color of shells is heritable [26], and the inner shell color is a breeding target that can improve breeding efficiency [27]. The addition of small pieces of mantle with different inner shell colors will have a significant impact on the color of the pearls produced [28, 29]. Compared with traditional breeding methods, molecular marker-assisted breeding as an emerging breeding method can greatly improve breeding efficiency [30] and has been studied in a variety of aquatic animals [31–33]. In this experiment, primers were designed using the known full-length cDNA sequence of *PKC* in the *H. cumingii*. After primer amplification and sequencing, five SNP sites were found in the exons of the *HcPKC* gene, which was significantly higher than the 1SNP/1000 bp in the previous study [34]. This indicates that there are relatively abundant single nucleotide polymorphisms in the *HcPKC* gene. According to the polymorphism analysis, it was found that in the *HcPKC* gene, the A + 332G site is a moderate polymorphism site, and the other four sites are low polymorphism sites, but no high polymorphism was found in this gene. This is because SNP markers are DNA sequence polymorphisms caused by single nucleotide variation, and it is difficult to show higher polymorphisms such as in Simple Sequence Repeat (SSR) markers [35]. Preliminary analysis of the SNP correlation between the purple and white inner shell color of the spinnaker mussels and the *HcPKC* gene showed that the genotypes of the G + 217 T locus had significant differences in *b* and *dE* parameters (*P* < 0.05), A + 332G. The genotypes of the loci were significantly different in *L*, *b* and *a*, *dE* parameters (*P* < 0.05). It is speculated that this gene may play a certain role in the formation of nacre color in the *H. cumingii* [36, 37]. Due to the limitation of the number of samples, this experiment can explain the problem to a certain extent, and subsequent experiments need to further expand the sample size to verify the results of this study.

To further investigate whether the polymorphism of the *HcPKC* gene is associated with nacre color traits, we analyzed linkage disequilibrium [38] and haplotype analysis [39]. The results showed that among the haplotypes constructed by the *HcPKC* gene, therefore, the dominant type can be selected according to demand to speed up breeding efficiency and provide a reference for the rapid selection of the target shell color [40].

## Conclusions

In this study, a *HcPKC* gene was fully cloned in *H. cumingii* and investigated for the first time. Validation of the effect of *HcPKC* gene on shell nacre by fluorescence quantification, in situ hybridization experiments, and discovery of single-nucleotide polymorphisms (SNPs) associated with inner shell color-related traits that *HcPKC* of *H. cumingii* can be used as a candidate gene related to inner shell color, and some of the SNP sites can be used for molecular-assisted breeding in the spinnaker mussel, providing a reference for cultivating high-quality freshwater pearls.

## Methods

### Ethical approval statement

*H. cumingii* were treated according to animal care and use guidelines for scientific purposes established by the Institutional Animal Care and Use Committee of Shanghai Ocean University, Shanghai, China.

### Experimental materials

**Fig. 4:**
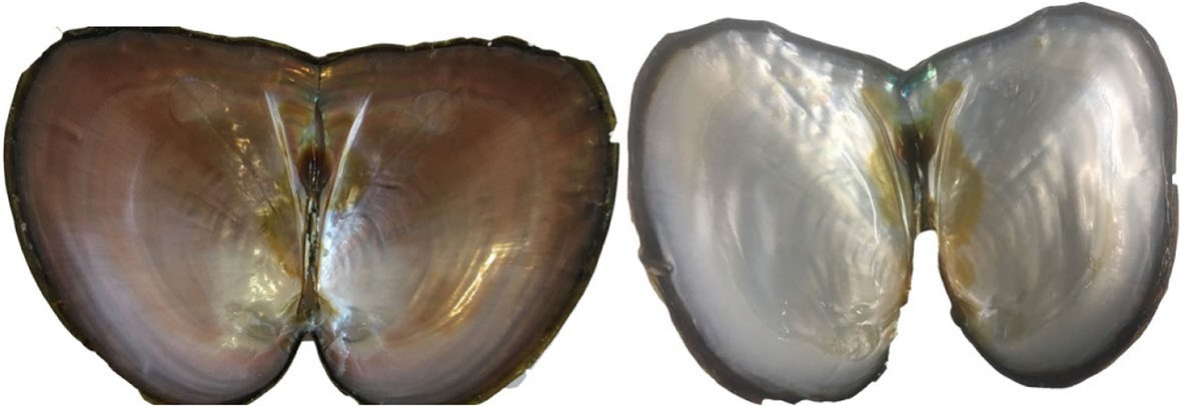
Purple (left) and white (right) H.cumingii mussels used in the experiment

Two-year-old healthy *H*. *cumingii* mussels (average shell length of 10 cm) with purple and white inner-shell colors were obtained from Weimin Aquaculture Base, Jinhua City, Zhejiang Province, China (Fig. 4). Before the experiment, the mussels were placed in a laboratory water tank for oxygenation for about a week, and then fresh mantle samples were stored at − 80 °C for later use.

### Experimental method

#### Total RNA extraction and cloning of the full-length *HcPKC*

The TRIzol method was used to extract total RNA from healthy mantle tissue samples. The SMARTer RACE 5′/3′ kit was used to synthesize RACE-Ready cDNA as a gene cloning template. Specific primers (Table 5) were designed based on the *HcPKC* (*HcPKC*-F and *HcPKC*-R) expressed sequence tags (ESTs) of *H. cumingii* which were obtained from the *H. cumingii* mantle transcriptome library [41]. The PKC gene fragment was obtained from a mantle transcriptome library of *H. cumingii* (Table 5), and the specific primers were designed by Primer 5.0 to perform PCR amplification and verify the sequence. According to the SMARTer RACE 5′/3′ kit instructions, 5′-RACE and 3′-RACE specific primers were designed, RACE cloning was performed, and the DNA was sequenced by Sangon (Shanghai, China) to obtain the full-length PKC gene.

**Table 5:** Primers used in the study

primers	(5′-3′)sequence of primers	purpose
*HcPKC*-F	GCTTGTTTCCAGACTGACGA	Partial fragment amplifification of *HcPKC*
*HcPKC*–R	CGTCAGTCTGGAAACAAGCAA	Partial fragment amplifification of *HcPKC*
*HcPKC*-3’	CCATCCATTCCTTGTAAACCTG	3’RACE
*HcPKC*-5’	GTCAGTCTGGAAACAAGCAAAC	5’RACE
HcPKC-RT-F	AGTGAACGTGATGCAGAGGA	qPCR
HcPKC-RT-R	GGTGTCAACACTGGCTTCTC	qPCR
HcPKC-Y-F	AGTGAACGTGATGCAGAGGA	In situ hybridization
HcPKC-Y-R	TAATACGACTCACTATAGGGGGTGTCAACACTGGCTTCTC	In situ hybridization
EF1α-F	GGAACTTCCCAGGCAGACTGTGC	qPCR internal control
EF1α-R	TCAAAACGGGCCGCAGAGAAT	qPCR internal control

#### Gene sequence analysis

ORF Finder (https://www.ncbi.nlm.nih.gov/orffinder/) was used to predict the ORF (open reading frame) of the *HcPKC* gene sequence and the encoded amino acid sequence [42]. Smart Blast was used to predict amino acid sequence homology analysis [43]. The amino acid inclusion domains were analyzed by Simple Modular Architecture Research Tool SMART (http://smart.embl-heidelberg.de/). The Protparam online tool (https://www.expasy.org/) was used to obtain information on physical parameters such as amino acid sequence composition, molecular weight, isoelectric point, etc. [44]. ClustalX software was used for multiple sequence alignment analysis [45] and MEGA 5.2 (Arizona State University, USA) was used to construct a phylogenetic tree [46].

#### Tissue-specific expression analysis of the *HcPKC* gene

Hepatopancreas, gill, adductor muscle, foot, marginal membrane, central membrane samples were taken from six healthy *H*. *cumingii* individuals and were used for RNA extraction. The RNA was then reverse-transcribed to cDNA by using SYBR®Premix Ex Taq II (TliRNaseH Plus, TaKaRa). Bio-Rad-CFX-96 (Bio-Rad, USA) was used for fluorescence quantitative PCR. The PCR reaction mixture was as follows: SYBR®Premix Ex Taq II (TliRNaseH Plus), 10 μL; upstream and downstream primers, 0.8 μL; ddH_2_O, 6.8 μL and cDNA template 1.6 µL. Each reaction was performed in three replicates. The reaction parameters were: pre-denaturation at 95 °C for 30 s; followed by 40 cycles of 95 °C for 5 s; 56 °C for 35 s; and 72 °C for 30 s. Referring to the previous research results of our laboratory, EF1α was used as an internal reference gene [47] (Table 5).

#### In situ hybridization

Specific primers were designed and the T7 promoter sequence TAATACGACTCACTATAGGG (Table 5) was added at the 5′ end. The target fragment was obtained after PCR amplification and product purification, and in vitro transcription was performed using a Complete Gold in vitro transcription kit. The fresh mantle tissue of the mussel was placed in 4% paraformaldehyde to fix and dehydrate for 4 h (in a 4 °C refrigerator), then placed in 25% sucrose solution at 4 °C overnight. The tissue was cut into ~ 10 μm sections. They were marked and stored on glass slides at − 80 °C for later use. Follow-up in situ hybridization experiments were performed later.

#### Extraction of genomic DNA

For SNP experiments, 70 white mussels and 70 purple mussels were selected randomly. The genomic DNA of the experimental samples was extracted using a TIANamp Marine Animals DNA Kit and coagulated with 1% agarose. The quality of DNA was detected by gel electrophoresis and a NanoDrop 2000C spectrophotometer, and the samples were placed in a − 20 °C refrigerator for later use.

#### Data measurement

Using a Lovibond-RT200 surface colorimeter to measure the inner shell color of purple and white experimental mussels, and according to the uniform color space determined by the International Commission on Illumination (CIE), L* represents the brightness. L* > 0 indicated that the color was bright, L* < 0, darker color; a* > 0, redder color, a* < 0, greener color; b* > 0, yellowish color, and b* < 0, bluer color [48]. The anterior, middle, and posterior margins of the right shell of 140 mussels were measured, and the difference in the color parameter was calculated as follows: *dE* = (L^2^ + a^2^ + b^2^)^½^, *L* = *L*x-*L*0, a = *a*x-*a*0, b = *b*x-*b*0. *L*x, *a*x, and *b*x are the color parameter values of different shells. *L*0, *a*0, and *b*0 are the color parameters of standard white inner shell mussels and M*L*, M*a*, M*b*, and M*dE* represent the average value of *L*, *a*, *b*, and *dE*.

#### Screening of SNP loci in the *HcPKC* gene of *H. cumingii*

The *HcPKC* gene was compared with the *PKC* gene in the genome of the *H. cumingii* to determine the exon and intron regions. Primers specific to exonic regions were designed (Table 6). The DNA samples of 10 white mussels and 10 purple mussels were selected randomly for sequence amplification, and the amplified products were sent to MAP BIOTECH (Shanghai) for sequencing. Sequence 5.4.6 was used to obtain the SNP site from the compared sequencing results.

**Table 6:** The primers of SNP in the *HcPKC* gene of *H. cumingii*

primers	(5′-3′)sequence of primers
F1	CTTTATTGACAATGGCAGAGCA
R1	AGTTCTGCTAAACCCCTCCAT
F2	TAACCATGATGATTTGTCTTCCTCT
R2	TTCCAGCAAACAGGACTGATTAT

#### Data analysis

Genetic parameters such as observed heterozygosity, expected heterozygosity, and polymorphism content were analyzed using Popgene software [49]. The chi-square test was performed using SPSS software to analyze the correlation between the genotypes of different SNPs in the *HcPKC* gene fragment and the inner shell color of the mussels [50]. Analysis of linkage disequilibrium and haplotype construction with SHEsis online software (http://analysis.bio-x.cn/) [51, 52].

#### Statistical analysis

Data are shown as the mean ± SD and was analysed using SPSS 17.0 software. Differences were recognized as significant when *p* < 0.05 and highly significant when *p* < 0.01.

## Data Availability

All data generated during this study are included in this published article.

## Abbreviations

PKC: Protein kinase C
SNP: Single-nucleotide polymorphisms
H. cumingii: Hyriopsis cumingii
HcPKC: A PKC gene in Hyriopsis cumingii
